# Serotonin-Mediated Tuning of Human Helper T Cell Responsiveness to the Chemokine CXCL12

**DOI:** 10.1371/journal.pone.0022482

**Published:** 2011-08-10

**Authors:** Elena Magrini, Ildikò Szabò, Andrea Doni, Javier Cibella, Antonella Viola

**Affiliations:** 1 Humanitas Clinical Institute IRCCS, Rozzano, Milan, Italy; 2 Department of Biology, University of Padua, Padua, Italy; 3 Department of Translational Medicine, University of Milan, Rozzano, Milan, Italy; Rega Institute, University of Leuven, Belgium

## Abstract

In addition to its role as neurotransmitter, serotonin (5-HT) is an important modulator of inflammation and immunity. Here, we report novel findings suggesting a 5-HT involvement in T cell migration. In particular, we show that 5-HT tunes the responsiveness of human T lymphocytes to the broadly expressed chemokine CXCL12 in transwell migration assays. By real-time PCR, western blot analysis and electrophysiological patch clamp experiments, we demonstrate that the type 3 5-HT receptor (5-HT_3_) is functionally expressed in human primary T cells. In addition, specific 5-HT_3_ receptor agonists selectively decrease T cell migration towards gradients of CXCL12 but not of inflammatory chemokines, such as CCL2 and CCL5. In transmigration experiments, 5-HT_3_ receptor stimulation reverts the inhibitory effect of endothelial-bound CXCL12 on T cell migration. Our data suggest that the reduced T cell responsiveness to CXCL12 induced by 5-HT may occur to facilitate T cell extravasation and migration into inflamed tissues.

## Introduction

The monoamine serotonin (5-HT) is known to mediate a plethora of actions throughout the body. In the central and autonomous nervous system, 5-HT is utilized by neurons to signal both volumic and fast synaptic neurotransmission. In the periphery, 5-HT can both contract and relax smooth muscle (vascular and gastrointestinal), contract cardiac muscle, regulate hepatic microcirculation and contribute to blood clotting [1].

Synthesis of 5-HT in the peripheral and central nervous system is respectively operated by two different isoforms of tryptophan hydroxylase (TPH), TPH-1 and -2 [2]. These enzymes catalyze hydroxylation of tryptophan to 5-hydroxytryptophan, the immediate precursor of 5-HT. Though conventionally considered a neurotransmitter, 5-HT is primarily produced by the enterochromaffin cells in the gut [3] and it is taken up *via* an active transport mechanism into platelets, which provide a rich reservoir of 5-HT in the circulation. Subsequent release of platelet-stored 5-HT can be rapidly triggered by platelet-activating factor, thrombin, complement fragments C3a and C5a, or immunoglobulin E-containing immune complexes. Thus, at sites of inflammation and platelet activation, local concentrations of 5-HT can greatly exceed the relatively low amounts found free in the serum [4]. Indeed, 5-HT is a known immunoregulator and it is implicated in several inflammatory diseases [1], [5]–[7]. In the context of the adaptive immune response, 5-HT affects both T cell and dendritic cell functions [6], [8]. Interestingly, it has been recently reported that dendritic cells uptake 5-HT at sites of inflammation and shuttle it to naive T cells, thereby modulating T-cell activation [9], proliferation and differentiation [10].

The 5-HT receptors have been divided into seven major classes (5-HT_1_–5-HT_7_). The 5-HT_3_ receptor is the most phylogenetically conserved; it is described as a ligand-gated ion channel, permeable to monovalent and divalent cations [11], belonging to the Cys-loop receptor super-family and sharing structural and functional features with other members of this channel/receptor group [12]. The 5-HT_3_ receptor is constituted of five subunits organized in a channel. To date, five different 5-HT_3_ receptor subunits were identified (5-HT_3A–E_) and, among them, the 5-HT_3A_ subunit is essential to form a functional receptor, whereas the 5-HT_3B_ subunit is known to alter the pharmacological properties of the channel [13], [14].

In this study, we analyzed the possible role of 5-HT in T cell migration. We found that 5-HT selectively inhibits migration towards CXCL12 through the engagement of 5-HT_3_ receptor. We propose that this process likely facilitates T cell infiltration in inflamed tissues.

## Results

### 5-HT_3_ receptor activity modulates CXCL12-induced T cell migration

**Figure 1:**
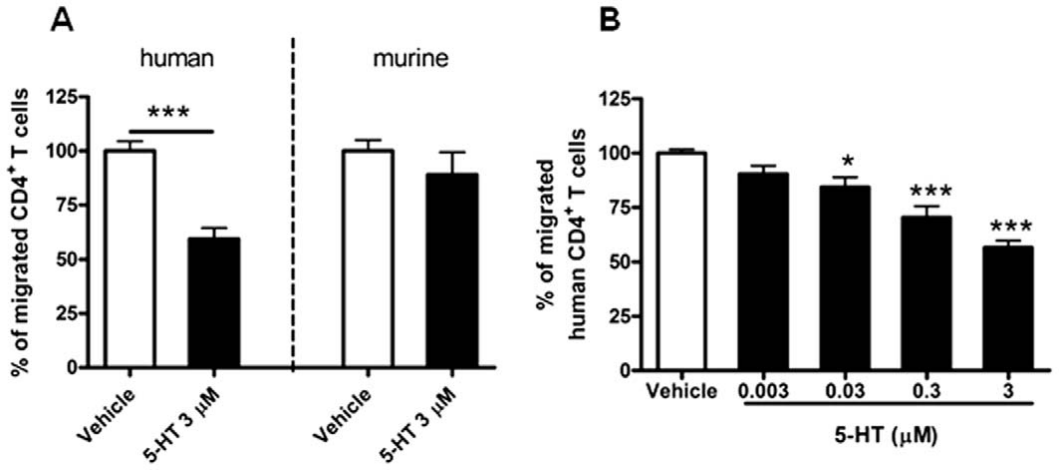
5-HT reduces human CD4^+^ T cell migration towards CXCL12 in a dose-dependent manner. Human primary (n = 3, Panel A; n = 3, Panel B) or murine naive (n = 3, Panel A) CD4^+^ T cells were treated with the indicated concentrations of 5-HT for 30 min at RT and then allowed to migrate towards CXCL12. Number of migrated human CD4^+^ T cells resulted 1232±354 compared to 685±179 for untreated and 5-HT-treated cells, respectively (mean ± SEM; Panel A). Percentages of migrated cells for each condition were calculated as described in “Materials and Methods”. Data were statistically analyzed with either *t*-test (A) or Bonferroni's multiple comparison test (B); *P<0.05, ***P<0.001.

First, we investigated on the possible modulation of human and mouse CD4^+^ primary T cells chemotaxis by 5-HT. The cells were treated with 5-HT and allowed to migrate in a transwell assay towards a gradient of CXCL12, a chemokine that binds and triggers the chemokine receptor CXCR4. After stimulation with 5-HT, human CD4^+^ T cells displayed decreased migration, whereas the migration of mouse CD4^+^ T cells was not affected (Figure 1A). The inhibition of CXCL12-induced human T cell migration exerted by 5-HT was dose-related in the range from 0.003 to 3 µM (Figure 1B).

**Figure 2:**
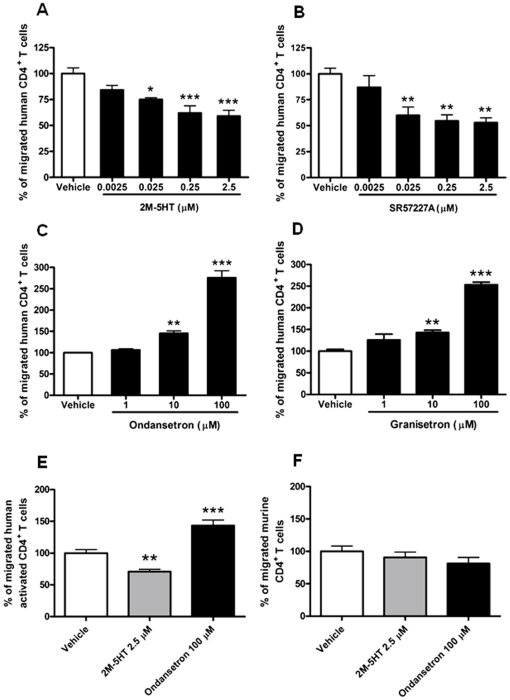
5-HT_3_ receptor activation reduces CXCL12-induced migration of resting and activated CD4^+^ T cells. Human resting (A–D), human activated (E) or murine naive (F) CD4^+^ T cells were treated with the indicated concentration of 5-HT_3_ receptor agonists, 2M-5HT (A) and SR57227A (B), or antagonists, ondansetron (C) and granisetron (D), for 30 min at RT and then used for migration assays towards CXCL12 (n = 3, from Panel A to Panel D); number of human activated CD4^+^ T cells migrated in control condition resulted 6102±1033 compared to 4559±846 and 8206±1407 for 2M-5HT and ondansetron treated cells, respectively (mean ± SEM, n = 4, Panel E); (n = 3, Panel F). Percentages of migrated cells for each condition were calculated as described in “Materials and Methods”; *P<0.05, **P<0.01, ***P<0.001, Bonferroni's multiple comparison test.

The 5-HT_3_ receptor subtype is expressed in human but not mouse T cells [9], [15] and thus we hypothesized that different effects of 5-HT on human and mouse T cell migration might depend on its specific expression. We evaluated T cell migration after incubation with specific 5-HT_3_ receptor agonists, SR57227A and 2-methyl-5-hydroxytryptamine (2M-5HT). As shown in Figures 2A and 2B, 5-HT_3_ agonists reduced, in a dose-dependent manner, CXCL12-induced migration of human resting T cells. We observed that 5-HT_3_ receptor agonists and 5-HT had a similar inhibitory effect on T cell chemotaxis, suggesting that the effects of 5-HT on CXCL12-induced cell migration were most likely mediated by 5-HT_3_ receptor engagement. Accordingly, the two selective 5-HT_3_ receptor antagonists enhanced T cell chemotactic response to CXCL12 in the range from 1 to 100 µM (Figure 2C–D). Similar results were obtained when human T cells activated by anti-CD3 and anti-CD28 antibodies (Abs) were used (Figure 2E). The use of different chemicals, including 5-HT, did not alter neither spontaneous chemotaxis nor modified T cell chemokinesis (not shown). In addition, in line with the absence of the receptor, 5-HT_3_ receptor agonist and antagonist did not affect the migration of mouse T cells (Figure 2F).

### Human T cells express functional 5-HT_3_ receptor and produce 5-HT

**Figure 3:**
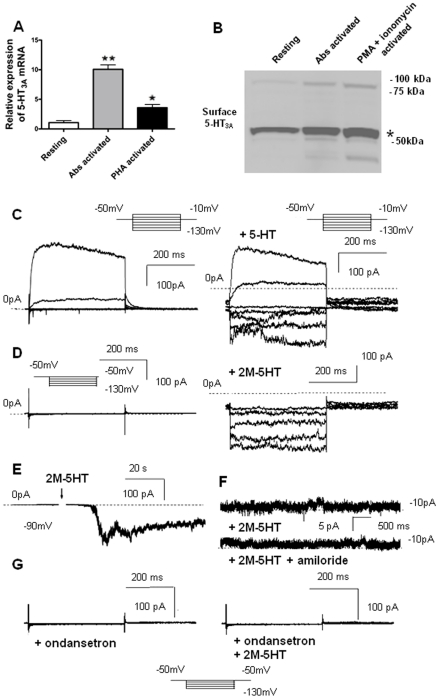
Resting and activated human CD4^+^ T cells express functional 5-HT_3_ receptor. Human CD4^+^ T cells were stimulated with anti-CD3 and anti-CD28 Abs, PHA or PMA plus ionomycin for 72 h. (A) Gene expression for 5-HT_3A_ receptor subunits was examined by real-time PCR. The graph represents the quantitative analysis of the experiments. Values are expressed as fold increase over resting T cells. Data are mean of triplicates ± SD of the 2^−ΔΔCt^ (n = 3); *P<0.05, **P<0.01, *t*-test. (B) T cell membranes were isolated by ultracentrifugation from total cell lysates and 20 µg/sample were separated on SDS-PAGE gel. A representative immunoblot of three experiments performed showing the expression of 5-HT_3A_ subunit is reported. Asterisk indicates the band corresponding to 5-HT_3A_ monomer. (C–G) Freshly isolated CD4^+^ T cells were subjected to electrophysiological patch clamp experiments in the whole-cell configuration. Cells were held at resting potential (−50 mV) and voltage pulses of 400 ms duration were applied in 20 mV steps ranging from −130 to −10 mV. Currents were recorded before (C, left panel) and after (C, right panel) addition of 3 µM 5-HT to the bath solution. Addition of 5-HT induced an inward current at hyperpolarizing voltages. Activity of Kv1.3 resulting in outward potassium current was seen at depolarizing voltages −30 and −10 mV. Pipette: Kgluconate containing solutions; bath: Nagluconate containing solutions. In the same experimental conditions, with the exception that cesium was used in the pipette solution and NaCl in the bath solution (D–G), currents were also recorded before (D, left panel) and after (D, right panel) addition of 3 µM of specific 5-HT_3_ agonist 2M-5HT to the bath solution. Cesium blocked outward potassium currents and Kv1.3 activity was avoided by applying voltage pulses only from −130 to −50 mV in 20 mV steps. Addition of 3 µM 2M-5HT to a cell held at −90 mV holding potential in the whole-cell configuration caused a significant inward current within few seconds (E). Currents were recorded at −70 mV holding potential in outside-out patch after addition of 3 µM 2M-5HT (F, upper trace) and after addition of 100 µM amiloride, in the presence of 2M-5HT, to the same patch (F, lower trace). Amiloride did not reduce the current. Whole-cell currents were recorded in presence of ondansetron (100 µM) (G, left panel) and after addition of 3 µM 2M-5HT in the presence of 100 µM ondansetron (G, right panel). Applied voltages ranged from −130 to −10 mV. Data in Figures 3C–G are representative of at least five experiments in the same conditions giving similar results.

These results prompted us to verify and characterize the expression of functional 5-HT_3_ receptors in human CD4^+^ T cells. We employed real-time PCR analysis to evaluate the expression of 5-HT_3A_ and 5-HT_3B_ subunits in primary, resting T lymphocytes as well as in T cells activated by anti-CD3 and anti-CD28 Abs, or phytohaemagglutinin (PHA). Activation of CD4^+^ T cell was assessed by the up-regulation of activation surface markers, such as CD69 and CD25 (not shown). As shown in Figure 3A, both resting and activated human T cells expressed the 5-HT_3A_ receptor subunit. Moreover, the levels of 5-HT_3A_ transcript increased upon cell activation (Figure 3A). In contrast, no expression of the 5-HT_3B_ subunit was found (not shown). Similar results were obtained by Western blot analysis, which showed that the 5-HT_3A_ subunit was constitutively expressed by CD4^+^ T cells and that its expression was augmented upon cell activation (Figure 3B).

Next, we assessed the functional properties of the 5-HT_3A_ receptor in human CD4^+^ T lymphocytes through electrophysiological patch clamp experiments performed in freshly-isolated T cells in the whole-cell configuration (Figure 3C–G). In naive T cells, Kv1.3, a voltage-gated, depolarization-activated potassium channel, is responsible for maintenance of the resting membrane potential (around −50 mV) [16]. In accordance, in primary T lymphocytes we recorded Kv1.3-mediated potassium outward currents, displaying typical Kv1.3 inactivation kinetics, at depolarizing voltages (Figure 3C, left panel) but no current was observed at hyperpolarizing voltages. Upon addition of 5-HT to the bath, an inward current appeared at negative voltages, indicating that 5-HT alters ionic currents in T cells (Figure 3C, right panel). In the experiments shown in Figure 3C, both pipette and bath solutions contained gluconate, which did not permeate chloride channels. Therefore, the outward current was due to the exit of potassium via Kv1.3, while the 5-HT-elicited inward current was due to the entrance of cations into the cell. Although 5-HT_3A_ homomers also allow calcium permeation, in our experimental setting the concentration of calcium in the extracellular solution was too low (1 mM) to give rise to a significant current [16], suggesting that the major charge carrying ion was sodium. In line with this, replacing sodium with N-methyl-D-glucamine ions, which did not permeate through sodium channels and 5-HT_3_ receptors, resulted in no agonist-induced inward current (not shown). Next, to eliminate signals due to Kv1.3 activity, in the following experiments we applied hyperpolarizing potentials (≤−50 mV) (Figure 3D–G) and cesium - a well-known blocker of potassium channels - was the only cation present in the pipette solution. Under these ionic conditions, the addition of 2M-5HT to resting T cells, which did not display any current at negative potentials under control condition (Figure 3D, left panel), elicited activation of negative, inward currents (Figure 3D, right panel). Moreover, in another representative experiment, addition of 2M-5HT to cells at −90 mV holding potential in the whole-cell configuration caused a significant inward current within few seconds (Figure 3E). T cells are known to express amiloride-sensitive sodium channels [17]. To exclude the possibility that the current recorded after addition of 2M-5HT was due to sodium influx through amiloride-sensitive channels rather than 5HT_3_ receptors, currents were recorded either in the absence (Figure 3F, upper trace) or in the presence of amiloride (Figure 3F, lower trace). Amiloride did not reduce 2M-5HT-induced currents, indicating that, upon addition of the 5-HT_3_ receptor agonist, sodium did not enter the cells via classical amiloride-sensitive sodium channels. In accordance with this observation, 2M-5HT failed to induce inward currents in cells pre-treated with ondansetron (Figure 3G).

**Figure 4:**
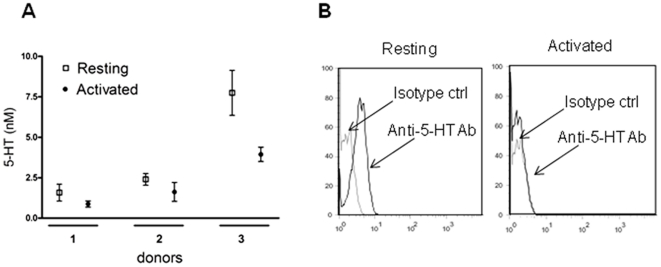
5-HT is produced and released by human CD4^+^ T cells. (A) Cell-released 5-HT was quantified by EIA. Data are mean of triplicate ± SEM (n = 3). (B) Representative flowcytometric analysis of three experiments performed showing intracellular 5-HT in primary and activated human T cells (MFI, normalized to isotype control staining, was 70.5±3.5 and 3±1 for resting and activated T cells, respectively; n = 3, *P*<0.01).

Human T cells were found to express TPH-1 [18], thus we asked whether T cells were able to synthesize 5-HT. To this end, we performed an Ultra-Sensitive Serotonin enzyme immunoassay (EIA) of supernatants obtained from resting and activated T cell cultured in medium supplemented with fetal calf serum (FCS) that was previously treated with charcoal-coated dextran, in order to absorb the endogenous 5-HT. Consistently with published data [9], complete media supplemented with 10% FCS contained 276±31 nM 5-HT, and this concentration was reduced to 1.1±0.3 nM following treatment with charcol-coated dextran (mean ± SEM, n = 3). Despite the intra-donor variability, in all experiments performed we measured nanomolar concentrations of 5-HT released by both resting and activated cells (Figure 4A). However, the 5-HT release was reduced upon T cell activation (*P*<0.01). These observations were confirmed by intracellular staining and flow cytometric analysis (Figure 4B).

### 5-HT_3_ receptor stimulation enhances T cell transmigration through CXCL12-coated endothelial cells

**Figure 5:**
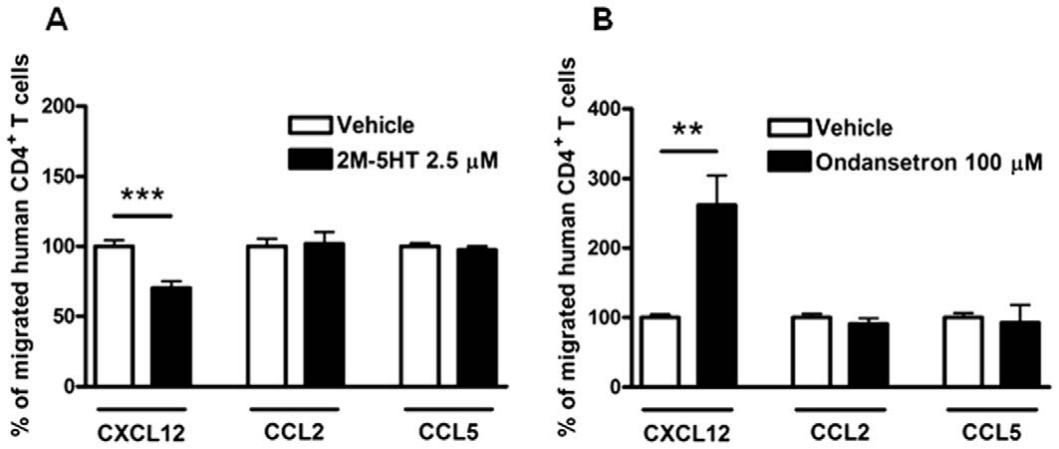
5-HT_3_ receptor activity selectively modulates CXCL12-induced chemotaxis of human CD4^+^ T cells. Human resting CD4^+^ T cells were treated with 2.5 µM 2M-5HT (n = 3, Panel A) or 100 µM ondansetron (n = 3, Panel B) for 30 min at RT, and then allowed to migrate towards CXCL12, CCL2 or CCL5. Percentages of migrated cells for each condition were calculated as described in “Materials and Methods”. **P<0.01, ***P<0.001, *t*-test.

We asked whether 5-HT modulates chemotactic responses towards chemokines other than CXCL12. Thus, we evaluated the effects of 5-HT_3_ receptor agonist (Figure 5A) and antagonist (Figure 5B) on human T cell migration towards CCL2 or CCL5 gradients. Both 2M-5HT and ondansetron had no effect on T cell migration towards the inflammatory chemokines CCL2 or CCL5, indicating that the 5-HT/5-HT_3_ system did not alter T cell chemotactic responses to all chemokines and did not act on general mechanisms required for migration.

**Figure 6:**
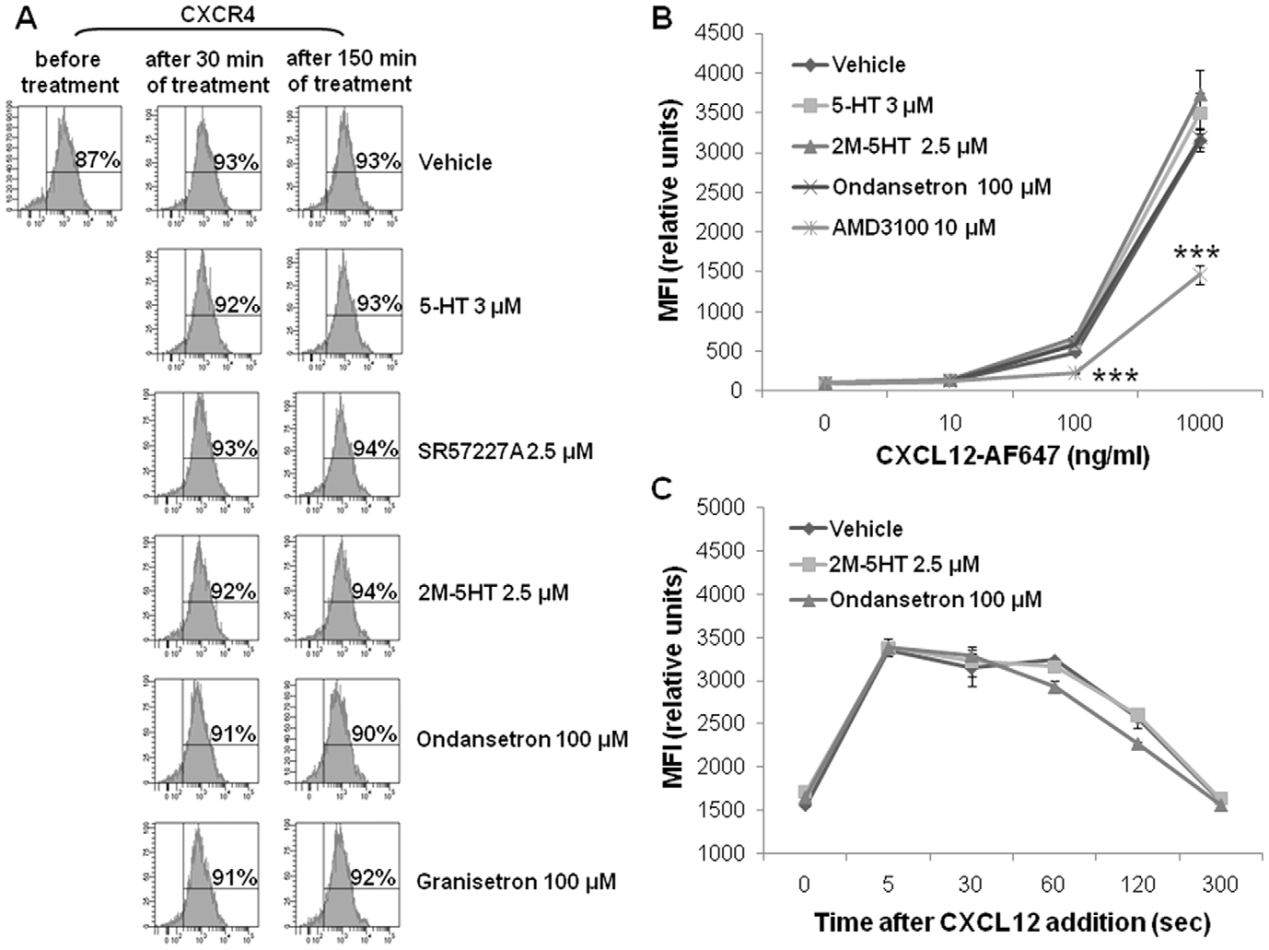
5-HT_3_ receptor activity does not modify CXCR4 expression, CXCL12 binding and CXCL12-induced actin polymerization. (A) After treatment of resting human CD4^+^ T cells with 5-HT, the 5-HT_3_ receptor agonist 2M-5HT or the 5-HT_3_ receptor antagonist ondansetron for 0, 30 and 150 min, CXCR4 expression was analyzed by flow cytometry. Data refer to percentage of a representative experiment of three performed. (B) Binding of CXCL12-AF647 on human CD4^+^ T cells. The cells were treated for 30 min at RT with 5-HT, 2M-5HT, ondansetron or the specific CXCR4 antagonist AMD3100 (ref. 20). The cells were then incubated with increasing concentrations of fluorescent CXCL12, in the presence of the indicated chemicals. Data are expressed as MFI ± SEM vs. CXCL12-AF647 concentrations (n = 4; ***P<0.001, Bonferroni's multiple comparison test). (C) Human CD4^+^ T cells were treated with 2M-5HT or ondansetron for 30 min at RT and then stimulated with CXCL12. Kinetics of CXCL12-induced F-actin formation were analyzed by phalloidin staining and flow cytometric analysis. Data represent MFI ± SEM vs. time (n = 3).

In order to investigate the mechanism of 5-HT effect on CXCL12/CXCR4 response, we evaluated CXCR4 expression, CXCL12 binding and CXCL12-induced actin polymerization in T cells after stimulation or inhibition of 5-HT_3_ receptors. As shown in Figure 6, neither 5-HT nor 5-HT_3_ receptor agonists or antagonists had any detectable effect on CXCR4 expression (Panel A), CXCL12 binding to its receptor (Panel B) or CXCR4-mediated early signaling (Panel C).

**Figure 7:**
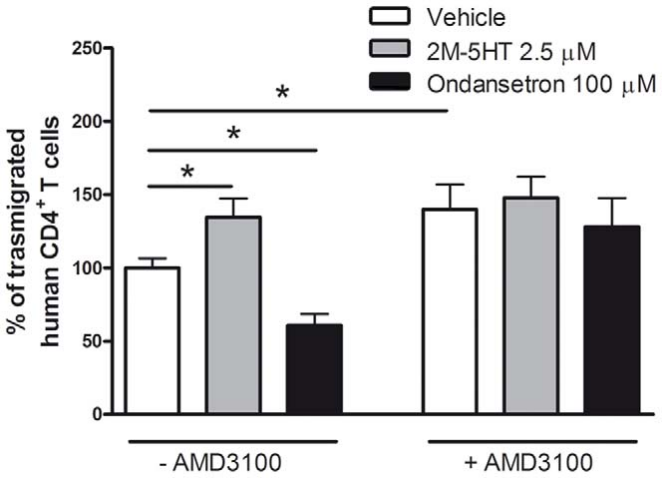
5-HT_3_ receptor activation enhances T cell transmigration through CXCL12-coated endothelial cells. Human resting CD4^+^ T cells were treated with 2.5 µM 2M-5HT or 100 µM ondansetron for 30 min at RT. Then, CD4^+^ T cells were incubated (+) or not (−) with the CXCR4 antagonist AMD3100 (10 µM) and immediately used for a transendothelial migration assay towards CCL2 through CXCL12-coated endothelial cells. In absence of AMD3100, number of migrated control cells resulted 1524±270 compared to 1888±252 and 1234±398 for 5-HT and ondansetron treated cells, respectively (mean ± SEM). Percentages of migrated cells for each condition were calculated as described in “Materials and Methods”. n = 5, *P<0.05, Bonferroni's multiple comparison test.

CXCL12 is present on the surface of endothelial cells [19] and thus we evaluated if 5-HT might influence T cell extravasation, by regulating endothelial-bound CXCL12-chemoattraction. To address this question, we compared the ability of human T lymphocytes, treated or not with the specific CXCR4 antagonist AMD3100 [20], to migrate towards an inflammatory stimulus, such as CCL2, through CXCL12-coated endothelial cells. Endothelial-bound CXCL12 had an inhibitory effect on T cell transendothelial migration and, interestingly, 5-HT_3_ receptor stimulation by 2M-5HT enhanced transendothelial migration of T cells, whereas 5-HT_3_ receptor inhibition through ondansetron strongly reduced it (Figure 7). All these effects were specifically determined by the presence of endothelial-bound CXCL12, as they were abrogated by AMD3100 (Figure 7). Moreover, they did not depend on the presence of CCL2, which was simply used as a chemoattractive stimulus, given that 2M-5HT or ondansetron exhibited no effect on migration towards this chemokine, as previously demonstrated (Figure 5).

## Discussion

The attraction of leukocytes to the sites of inflammation and infection is an essential component of the host response to diseases. The recruitment of circulating leukocytes from the blood stream into inflamed tissues is driven by chemokines, produced either by infiltrating inflammatory cells or by injured tissue cells. This process depends on three distinct phases: vascular attachment of leukocytes to the luminal side of blood vessels, extravasation (transendothelial migration) and cell migration.

Platelets, at the sites of inflammation, play a crucial role as amplifiers of leukocyte adhesion to the endothelium because of their repertoire of adhesion molecules and soluble mediators [21]. 5-HT, one of the soluble mediators locally released by platelets, is emerging as a key regulator of immunity, tuning in a very complex way both innate and acquired responses [3], [6], [22]. In agreement, 5-HT seems to be directly involved in the pathogenesis of several inflammatory diseases, including irritable bowel syndrome [23], colitis [24], contact allergy [25], atopic dermatitis [26], psoriasis [27] and asthma [28]. Moreover, recent studies have demonstrated that platelet-derived 5-HT aggravates T-cell induced liver damage in viral hepatitis [29], [30].

The data reported in this study indicate that 5-HT inhibits migration of human T cells towards CXCL12 gradients and that this effect depends on the activity of 5-HT_3_ receptors, which are functionally expressed in human T cells.

The presence of functional 5-HT_3_ receptor has been already described in the Jurkat T cell line using the fluorescent probe SBFI/AM [31]. Here, through the patch clamp technique, we show the presence of 5-HT_3_ receptor agonist-induced inward currents, which are abolished by pre-incubation with 5-HT_3_ receptor antagonist, in human peripheral blood T cells, demonstrating for the first time that 5-HT_3_ receptors are also active in human primary T cells.

Interestingly, our data indicate that T cells produce 5-HT and suggest that they use it as an autocrine factor to reduce responsiveness to CXCL12. Indeed, all our chemotaxis experiments have been performed in serum-free conditions and hence in the absence of external or contaminant sources of 5-HT. In these conditions, the boosting effect of 5-HT_3_ receptor antagonists on T cell migration suggests - but does not formally prove - that self-produced 5-HT controls responsiveness to CXCL12. In addition, T cells are able to sense an increase in the concentration of extracellular 5-HT, and respond with a further reduction of responsiveness to CXCL12 stimulation.

One puzzling aspect of our findings concerns the molecular basis for the selective action of the serotonin receptor 5-HT_3_ on CXCL12/CXCR4. A trivial explanation for the selective control of CXCL12-induced migration may be a direct modulation of the expression of CXCR4 - the CXCL12 receptor - by 5-HT. However, CXCR4 expression and its affinity for the chemokine are not altered by 5-HT or by 5-HT_3_ receptor agonists or antagonists. Moreover, 5-HT_3_ receptor stimulation or inhibition do not alter actin polymerization in response to CXCL12, indicating that the early events following CXCR4 stimulation are not affected by 5-HT. The mechanism responsible for the specific modulation of CXCL12-induced T cell migration by the 5-HT system is therefore still unclear and may involve pH_i_ regulation. Chemotaxis is indeed modulated by the contribution of several different ion channels and by pH_i_ changes [32]–[35], although the precise mechanisms are still not clear.

CXCL12 regulates both homeostatic and pro-inflammatory migration and homing of lymphocytes [36]–[38] and we suggest that the selective control of T cell responses to CXCL12 by 5-HT may be important in these contexts. Notably, it has been previously observed that the neutrophil elastase cleaves CXCL12 expressed on the bone marrow stroma, thus facilitating stem cell mobilization from bone marrow into the peripheral circulation [39], [40]. In addition, another study has shown that elastase release by transmigrating neutrophils deactivates endothelial-bound CXCL12 and attenuates subsequent T lymphocyte transendothelial migration [41]. Our data indicate that an additional mechanism has evolved to selectively control responsiveness of lymphocytes to CXCL12. We suggest that, reducing responsiveness to endothelial-bound CXCL12 chemoattraction, 5-HT facilitates transendothelial T cell migration towards gradients of inflammatory chemokines released in inflamed tissues. Data obtained with 5-HT_3_ receptor antagonists do not exclude the possibility that T cells themselves may control their responsiveness to CXCL12 in an autonomous manner. On the other hand, since released local concentrations of 5-HT consequent to platelet activation at sites of damaged endothelium greatly exceeds the relatively low amounts produced by T cells, our straightforward interpretation is that during T cell transendothelial migration the major source of 5-HT is most likely produced by platelets. In addition, the fact that activated T cells produce lower amounts of 5-HT than resting ones but up-regulate expression of 5-HT_3_ receptor may indicate that, upon activation, T cells become more dependent on external sources of 5-HT released, for example, by platelets.

In conclusion, our data unveil a novel connection between 5-HT and chemokines that may play a crucial role during tissue damage and inflammatory responses.

## Materials and Methods

### Ethics statement

Human peripheral blood T (PBT) cells were isolated from Buffy coats, a by-product of the isolation of blood coagulation factors, obtained from healthy donors at the Centre for Blood Transfusions of the Desio or Padua Hospital. The Centres for Blood Transfusions collected blood donations after a written informed consent, and did not release to our laboratory any personal data of the blood donors. For all these reasons, our Institution did not require approval from the local ethics committee.

Procedures involving animals and their care conformed with institutional guidelines (authorisation n. 11/2006-A from the Italian Ministry of Health) in compliance with national (4D.L. N.116, G.U., suppl. 40, 18-2-1992) and international law and policies (EEC Council Directive 86/609, OJ L 358,1,12-12-1987; NIH Guide for the Care and Use of Laboratory Animals, US National Research Council 1996). All efforts were made to minimize the number of animals used and their suffering. Full details of the study were approved by Istituto Clinico Humanitas Animal Care and Use Committee (IACUC).

### Cell culture and activation

Human CD4^+^ PBT cells were isolated from the peripheral blood of healthy donors by negative selection using RosetteSep kit (StemCell Technologies) and cultured in RPMI 1640 medium (Lonza) supplemented with non essential amino acids,1 mM sodium pyruvate, 2 mM L-glutamine, 10 mM Hepes buffer (Lonza) (complete) and 10% FCS (Euroclone). In some experiments, human cells were activated by culture for 72 hours (h) in the presence of 2.5 µg/ml plate-bound functional grade purified anti-human CD3 Ab (OKT3; eBioscience) and 1 µg/ml of purified NA/LE anti-human CD28 Ab (CD28.2; BD Biosciences) in solution, with 2.5 µg/ml of PHA (Biochrom AG) or with a combination of 10 nM of phorbol 12-myristate 13-acetate (PMA; Sigma) and 1 µM of ionomycin (Sigma). T cell activation was determined by labeling with anti-human CD25 Ab (2A3; BD Biosciences) and anti-human CD69 Ab (FN50; BD Biosciences). The expression of CXCR4 was assessed by labeling with anti-human CD184 (CXCR4) Ab (12G5; BD Biosciences). Samples were analyzed using a FACS Canto flow cytometer and FACS Diva software (BD Biosciences).

Murine naive CD4^+^ T cells were obtained from the lymph nodes of C57BL/6 mice. Cells were sorted by negative selection using a mixture of rat anti-mouse CD8 (TIB105), CD19 (1D3), CD11b (A506) Abs and anti-rat IgG microbeads according to the manufacturer's instructions (Miltenyi Biotec). Murine CD4^+^ T cells were cultured in complete DMEM medium (Lonza) supplemented with 50 µM β-mercaptoethanol (Gibco) and 10% FCS. Percentage of CD4^+^ cells was determined by flow cytometric analysis after labeling with anti-mouse CD4 Ab (GK1.5; BioLegend).

Human umbilical vein endothelial cells (HUVECs) were gently gifted by M. Sironi (Milan, Italy) [42]. HUVECs were cultured in Medium 199 (Lonza) supplemented with 2 mM L-glutamine, 100 U/ml penicillin/streptomycin (Lonza), 100 µg/ml heparin (Sigma), 50 µg/ml EC growth supplement (ECGS; Biomedical Technologies, Inc.) and 20% FBS in flasks coated with 1% gelatine (Sigma) in PBS1× (Lonza) for at least 2 h at 37°C.

### Chemicals

5-HT, 2M-5HT, SR57227A, ondansetron hydrochloride dehydrate, granisetron hydrochloride, amiloride hydrochloride hydrate and AMD3100 were all purchased from Sigma. Stock solutions and intermediate dilutions of 5-HT and 5-HT_3_ agonists or antagonists were freshly prepared in water before each experiment and kept on ice protected from direct light.

### Real-time PCR

Total RNA from human CD4^+^ T cells was extracted using RNAspin Mini RNA isolation Kit (GE Healthcare) and treated with TURBO DNA-free Kit (Ambion). Total RNA from human dorsal root ganglion (hDRG; Clontech) was used as positive control. Reverse transcription was performed using the Superscript III First-Strand Synthesis System (Invitrogen). Real-time PCR was performed using SYBR Green PCR Master Mix (Applied Biosystems) and detected by 7900HT Sequence Detection System (Applied Biosystems). The following primers were used: human 18S (forward: 5′-CGCCGCTAGAGGTGAAATTC-3′ and reverse: 5′-CTTTCGCTCTGGTCCGTCTT-3′), human 5-HT_3A_ (forward: 5′-GCTTGCCAGAAAAGGTGAAATC-3′ and reverse: 5′-GGCGGATGACCACATAGAACTT-3′), human 5-HT_3B_ (forward: 5′-CCCTACCTCTAAGTGCCATCT-3′ and reverse: 5′-GGCTTATAGTTCTCAATGGTCCC-3′).

Data were analyzed using the SDS2.2.2 software (Applied Biosystems). The fold increase corresponded to 2^−ΔΔCt^, where the ΔC_t_ was the difference of the mean value of the C_t_ between the studied and the housekeeping gene (18S ribosomal RNA), and the ΔΔC_t_ was the difference of the ΔC_t_ between activated and resting cells for each gene. The standard deviation (SD) of the 2^−ΔΔCt^ was calculated applying the formula: SD of ΔΔC_t_ * 2^−ΔΔCt^ * ln2.

### Western blotting

Human CD4^+^ T cells were collected on ice-cold 50 mM Tris-HCl (pH = 7.5), 2 mM EDTA containing protease inhibitors (Complete EDTA-free protease inhibitor cocktail tablets; Roche) (hypotonic buffer). Nuclei were eliminated by centrifugation (300 *g*, 10 min, 4°C) and membranes were isolated by further centrifugation (48.000 rpm, rotor Type 90 Ti, 45 min, 4°C) and solubilized in hypotonic buffer containing 1% NP40 (Calbiochem). Total protein concentration was determined by DC Protein assay (Bio-Rad Laboratories). Specifically, 20 µg of total protein for each sample were diluted in XT sample buffer (Bio-Rad Laboratories) containing 10% β-mercaptoethanol (Bio-Rad Laboratories), boiled for 10 min and stored at −20°C until use. Western blots were immunolabeled using polyclonal rabbit anti-human 5HT_3_ receptor Ab (1 µg/ml; Imgenex) and secondary HRP–conjugated goat anti–rabbit IgG Ab (GE Healthcare). Membrane chemiluminescence was acquired using a ChemiDoc XRS system and the Quantity One – 4.6.1 software (Bio-Rad Laboratories).

### Patch clamp

Freshly isolated human CD4^+^ T lymphocytes were subjected to electrophysiological patch clamp experiments in the whole-cell configuration (or outside out patch in Figure 3F). Whole-cell currents were recorded with an EPC-7 amplifier (List) as previously described [43] and data were analyzed by using pCLAMP8 program set (Axon Instruments). Leak currents were not subtracted. The bath solution was composed of 156 mM Nagluconate, 1 mM CaCl_2_, 1 mM MgCl_2_, 10 mM HEPES (pH = 7.3) and the intracellular solution contained 134 mM Kgluconate, 1 mM CaCl_2_, 1 mM MgCl_2_, 10 mM EGTA, 10 mM HEPES (pH = 7.3) for experiments where Kv1.3 activity and 5HT_3_ activities were recorded. In some control experiments, to block inward cation current, in the bath solution Nagluconate was replaced with N-methyl-D-glucamine chloride (NMDGCl). Where indicated, intracellular Kgluconate was replaced with 134 mM CsCl (pH to 7.3 with CsOH) to block outward potassium current and the bath contained NaCl instead of Nagluconate.

### 5-HT detection

To quantify 5-HT, CD4^+^ T cells (5×10^6^ cells/ml) were incubated for 24 or 72 h at 37°C in complete RPMI 1640 medium supplemented with 10% charcoal/dextran-treated FBS, 0,1% (w/v) L-ascorbic acid (Labor Diagnostika Nord), 30 µM tranylcypromine (Sigma). FCS was pretreated with 0.25% (w/v) charcoal-coated dextran (Sigma) overnight at 4°C. 5-HT released into T cell culture supernatants was quantified using the Ultra-Sensitive Serotonin enzyme immunoassay (EIA; Labor Diagnostika Nord); same pretreated media was used as assay diluent. For intracellular staining cells were fixed with paraformaldehyde 4% for 15 min at 4°C and stained with monoclonal mouse anti-serotonin (5HT-H209; Dako) and AlexaFluor488-goat anti-mouse IgG (Molecular Probes) using the BD Cytofix/Cytoperm kit (BD Biosciences) according to the manufacturer's instructions. Mean of Fluorescence Intensity (MFI) was measured by FACS Canto flow cytometer and analyzed by Flow-Jo software.

### CXCL12 binding assay

T cells were washed once with Hanks' balanced salt solution containing 20 mM Hepes and 0.2% BSA (Sigma) at pH = 7.4 and resuspended in the same buffer. First, they were treated for 30 min at RT with the indicated chemicals and then incubated for 30 min at RT with different concentrations of CXCL12-AF647 (Almac Sciences) in the range from 0 to 1000 ng/ml. Thereafter, cells were washed twice in assay buffer and fixed in 1% paraformaldehyde in PBS. MFI was measured by FACS Canto flow cytometer and analyzed with FACS Diva software.

### Actin polymerization assay

After 5 hours of serum starvation in RPMI 1640 medium, T cells were treated for 30 min at RT with the indicated chemicals and then stimulated with CXCL12 (100 nM) at 37°C in a rotary shaker. At indicated time points, stimulation was stopped by adding 4% formaldehyde. Intracellular staining for F-actin was performed by AlexaFluor488–phalloidin (Molecular Probes) using the BD Cytofix/Cytoperm kit (BD Biosciences) according to the manufacturer's instructions. MFI was measured by FACS Canto flow cytometer and analyzed with FACS Diva software.

### Migration assays

Cell migration in response to chemokine was tested using Transwell assays (24-well plate with inserts, 3 µm pore/6.5 mm, TC-treated; Costar). Briefly, after 2 h of serum starvation in migration medium (serum-free RPMI 1640 supplemented with 0.1% BSA), 5-HT, 5-HT_3_ receptor agonists or antagonists were added for 30 min at RT to cell suspension and then cell were used for migration assays. 5-HT_3_ receptor agonists and antagonists were present during the migration in both transwell compartments. Human (1.5 nM) and murine (6.3 nM) CXCL12 were purchased from PeproTech; human CCL2 (11.5 nM) and CCL5 (12.8 nM) were purchased from R&D; chemokines were all diluted in migration medium. The number of living cells spontaneously migrating through the filter was determined after 2 h at 37°C by counting all the samples for the same time (1 min) at FACS Canto flow cytometer.

HUVECs were seeded at 15–20×10^3^/well and cultured to 90% confluence on 24-well transwell tissue culture inserts (24-well plate with inserts, 5 µm pore/6.5 mm, TC-treated; Costar) as previously described [42]. HUVECs were activated using IL-1β (Peprotech) at 10 ng/ml final concentration for 4 h at 37°C in HUVEC-culture medium and then incubated with CXCL12 20 ng/ml final concentration in migration medium for 15 min at 37°C. T cells were treated for 30 min at RT with the indicated chemicals, then incubated with AMD3100 10 uM and immediately allowed to migrate towards CCL2 (11.5 nM) for 30 min at 37°C. AMD3100, 5-HT_3_ receptor agonists and antagonists were present during the migration in both transwell compartments. Migration experiments displayed an inter-donor variability in the absolute number of migrating T cells, thus for each experiment migration data have been normalized considering the number of counted cells in control conditions as 100%.

### Statistical analysis

All experiments were performed at least three times. All results are expressed as mean ± the standard error of the mean (SEM). The statistical significance was calculated using a Student's *t*-test or a one-way ANOVA, Bonferroni's multiple comparison test (GraphPad Prism 4). Differences were considered significant if *P*<0.05.
